# PATIENTS’ PAIN AND THEIR SPOUSAL CAREGIVERS’ NEGATIVE AFFECT: THE MODERATING ROLE OF SELF-EFFICACY

**DOI:** 10.1093/geroni/igz038.2503

**Published:** 2019-11-08

**Authors:** Suyoung Nah, Lynn Martire, Christina Marini

**Affiliations:** The Pennsylvania State University, University Park, Pennsylvania, United States

## Abstract

Spousal caregivers of chronic pain patients may experience high levels of negative affect, perhaps in part because they regularly witness patients’ suffering. Yet, few studies have examined the relation between patients’ chronic pain and spousal caregivers’ negative affect. According to social cognitive theory, individuals’ self-efficacy may modulate how much negative affect they experience in response to stressful situations. The purpose of this study was to test the hypothesis that spousal caregivers would report higher levels of negative affect on days when patients experienced higher levels of knee pain. We also tested the hypothesis that patients’ and spouses’ self-efficacy for managing pain would each buffer this positive association. A total of 144 knee osteoarthritis (OA) patients and their spouses completed baseline interviews and a 22-day diary assessment. Multilevel models indicated that patients’ self-efficacy, but not spouses’ self-efficacy, moderated the positive association between patients’ pain and their spouses’ negative affect, even after controlling for spouses’ gender, age, and depressive symptoms. That is, spouses reported higher levels of negative affect on days when patients experienced more pain, but only among patients whose self-efficacy for managing pain was low. These findings suggest that patients’ self-efficacy for managing pain may serve as a protective factor for their spousal caregivers’ daily negative affect. Interventions targeting patients’ self-efficacy for managing pain may be beneficial for couples coping with knee OA.

